# Patterns of multimorbidity associated with 30-day readmission: a multinational study

**DOI:** 10.1186/s12889-019-7066-9

**Published:** 2019-06-13

**Authors:** Carole E. Aubert, Jeffrey L. Schnipper, Niklaus Fankhauser, Pedro Marques-Vidal, Jérôme Stirnemann, Andrew D. Auerbach, Eyal Zimlichman, Sunil Kripalani, Eduard E. Vasilevskis, Edmondo Robinson, Joshua Metlay, Grant S. Fletcher, Andreas Limacher, Jacques Donzé

**Affiliations:** 1 Department of General Internal Medicine, Bern University Hospital, University of Bern, Bern, Switzerland; 2 Institute of Primary Health Care (BIHAM), University of Bern, Bern, Switzerland; 3 BWH Hospitalist Service, Division of General Medicine, Brigham and Women’s Hospital, Boston, Massachusetts USA; 4 Harvard Medical School, Boston, Massachusetts USA; 5 CTU Bern, and Institute of Social and Preventive Medicine, University of Bern, Bern, Switzerland; 6 Department of Internal Medicine, Lausanne University Hospital, Lausanne, Switzerland; 7 Department of Internal Medicine, Geneva University Hospital, Geneva, Switzerland; 8 Division of Hospital Medicine, University of California, San Francisco, USA; 9 Sheba Medical Centre, Tel Hashomer, Israel; 10 Section of Hospital Medicine, Division of General Internal Medicine and Public Health Vanderbilt University, Nashville, TN USA; 11 Center for Clinical Quality and Implementation Research, Vanderbilt University, Nashville, TN USA; 12 VA Tennessee Valley, Geriatric Research, Education and Clinical Center, Nashville, TN USA; 13 Christiana Care Health System, Wilmington, Delaware USA; 14 Division of General Internal Medicine, Massachusetts General Hospital, Boston, USA; 15 Department of Medicine, Harborview Medical Center, University of Washington, Seattle, Washington USA; 16 Division of General Internal Medicine and Primary Care, Brigham and Women’s Hospital, Boston, MA 02120 USA

**Keywords:** Readmission, Potentially avoidable readmission, Multimorbidity, Diseases combinations

## Abstract

**Background:**

Multimorbidity is associated with higher healthcare utilization; however, data exploring its association with readmission are scarce. We aimed to investigate which most important patterns of multimorbidity are associated with 30-day readmission.

**Methods:**

We used a multinational retrospective cohort of 126,828 medical inpatients with multimorbidity defined as ≥2 chronic diseases. The primary and secondary outcomes were 30-day potentially avoidable readmission (PAR) and 30-day all-cause readmission (ACR), respectively. Only chronic diseases were included in the analyses. We presented the OR for readmission according to the number of diseases or body systems involved, and the combinations of diseases categories with the highest OR for readmission.

**Results:**

Multimorbidity severity, assessed as number of chronic diseases or body systems involved, was strongly associated with PAR, and to a lesser extend with ACR. The strength of association steadily and linearly increased with each additional disease or body system involved. Patients with four body systems involved or nine diseases already had a more than doubled odds for PAR (OR 2.35, 95%CI 2.15–2.57, and OR 2.25, 95%CI 2.05–2.48, respectively). The combinations of diseases categories that were most strongly associated with PAR and ACR were chronic kidney disease with liver disease or chronic ulcer of skin, and hematological malignancy with esophageal disorders or mood disorders, respectively.

**Conclusions:**

Readmission was associated with the number of chronic diseases or body systems involved and with specific combinations of diseases categories. The number of body systems involved may be a particularly interesting measure of the risk for readmission in multimorbid patients.

**Electronic supplementary material:**

The online version of this article (10.1186/s12889-019-7066-9) contains supplementary material, which is available to authorized users.

## Background

Multimorbidity, most often defined as the co-occurrence of two or more chronic diseases, is very frequent and affects 50 to 99% of hospitalized patients in Western countries [1–3]. Multimorbidity is strongly associated with age, and we may expect its prevalence to further increase in the coming years notably because of the rising life expectancy [4]. Hospital readmission within 30 days of discharge after an acute medical hospitalization is also frequent, affecting about 20% of the patients [5, 6]. Both multimorbidity and readmission have been associated with higher healthcare expenditures [3, 5–9].

A few studies have described an association between multimorbidity and readmission, but none looked at the potentially avoidable readmissions (PAR) specifically [7, 10, 11]. Furthermore, those studies measured multimorbidity mostly as a count of diseases, but the lack of standard to define which diseases should be included in this assessment limits generalizability of such analyses [12, 13]. Using validated indices or objective tools to categorize the diseases, such as the Chronic Condition Indicator (CCI) and the Clinical Classification Software (CCS) developed by the Healthcare Cost and Utilization Project, may improve comparability between studies [12–15].

Recently, growing interest has developed to assess non-random combinations of diseases among multimorbid patients [2, 3, 16–23]. However, little is known about how readmission is associated with such combinations of diseases, as well as with other measures of multimorbidity, such as the body systems involved. Furthermore, multimorbidity is a complex concept, with possible interactions between the different diseases leading to more or less than multiplicative effects on the risk for readmission, but this has never been assessed.

Using standardized tools to define chronic diseases and to classify them into clinically meaningful categories, the main objective of this study was to identify combinations of comorbidities associated with 30-day all-cause readmissions (ACR), and more specifically with 30-day PAR, in a large multinational retrospective cohort of multimorbid medical inpatients, to quantify this association, and to assess potential multiplicative effects of the diseases on the risk for readmission. The secondary aim was to quantify the association between readmissions and the number of chronic diseases and body systems involved.

## Methods

### Study design and population

We used a retrospective cohort including all multimorbid patients discharged home or to a nursing home from the medical inpatient wards of 11 large public hospitals (all but one academic) of three countries: the USA (7 hospitals), Switzerland (3 hospitals) and Israel (1 hospital) during calendar years 2010 and 2011. We defined multimorbidity as the presence of at least two chronic diseases. To categorize International Classification of Diseases (ICD) codes, we used the CCI and the CCS developed by the Healthcare Cost and Utilization Project, a Federal-State-Industry partnership sponsored by the Agency for Healthcare Research and Quality (AHRQ) [14, 15]. We included only chronic diseases according to the CCI and classified them into the 18 body system categories of the CCI (listed in Additional file 1) and into 285 exclusive diseases categories according to the CCS. For clinical relevance, we further merged some CCS categories and excluded ICD codes relating to risk factors, complications of diseases, symptoms or screening strategies (details in Additional file 1). All CCS categories (with categories numbers) found in the patients are listed in the Additional file 1. Reporting is in accordance with the STrengthening the Reporting of OBservational studies in Epidemiology (STROBE) statement [24]. All data were identified using electronic medical records. The dataset is not publically available, but it is available from the corresponding author on reasonable request (caroleelodie.aubert@insel.ch).

### Outcomes

Our primary outcome was PAR to any inpatient ward of the same hospital within 30 days following hospital discharge. PAR was defined by the SQLape algorithm, as previously described [25, 26]. Briefly, this algorithm classifies a readmission as unavoidable if it was foreseeable, for example for planned oncologic treatment, or if it involves a new body system not affected during the index admission. Conversely, treatment complications are classified as avoidable. Our secondary outcome was ACR to any inpatient ward of the same hospital. To avoid recording outpatient visits, we included only stays of at least 24 h.

### Statistical analyses

We presented baseline characteristics as median with interquartile range (IQR) for continuous variables and as numbers with frequencies for categorical variables. We performed a mixed-effects logistic univariable regression with a random intercept for center to account for correlation of the outcome data within the treating centers and presented the results as odds ratio (OR) with 95% confidence interval (CI) for PAR and ACR. We included only chronic diseases for all analyses. We considered following predictor variables in three distinct analyses: 1) count of diseases (2 to ≥10, reference = 2 diseases, as we included only patients with multimorbidity); 2) number of body system categories (1 to ≥7, reference = 1 body system category); 3) combinations of two diseases categories. For the latter, we presented the 20 combinations with the highest OR, comparing patients with to those without the combination, and assessed interactions between the diseases categories of each combination using the glmer function of the lme4 package in R, which implements generalized mixed models. We presented the results as either no significant interaction (0), more than multiplicative effect (+) or less than multiplicative effect (−). A more than multiplicative effect means that the two diseases categories in combination increased the odds for readmission more than just multiplying the odds for readmission of each respective disease category on its own. We performed all analyses with STATA 15.1 (StataCorp LP, College Station, TX, USA) or R version 3.4.4 (R Project for Statistical Computing).

## Results

From the 147,806 discharged patients available in the cohort, 126,828 (85.8%) were identified as multimorbid and included in the analysis, among which 12,203 (9.6%) had a PAR (Additional file 1: Figure S1) and 19,749 (15.6%) an ACR. Table 1 presents the baseline characteristics in relation to PAR. Median age was 64 years (IQR 52, 76) and median length of stay 5 days (IQR 3, 8). Median number of diseases was 6 (IQR 4, 9) in patients with PAR and 5 [3, 7] in those without.

**Table 1:** Baseline characteristics

Characteristics	Whole cohort (*N* = 126,828)	With PAR (*N* = 12,203)	Without PAR (*N* = 114,625)
Age, years	64 (52, 76)	63 (51, 75)	62 (52, 76)
Men	65,631 (51.7)	6481 (53.1)	59,150 (51.6)
Country			
Israel	10,020 (7.9)	1299 (10.6)	8721 (7.6)
Switzerland	33,871 (26.7)	1948 (16.0)	31,923 (27.8)
United States	82,937 (65.4)	8956 (73.4)	73,981 (64.5)
Description of Morbidity			
Number of chronic diseases	5 (3, 8)	6 (4, 9)	5 (3, 7)
Most frequent chronic diseases (prevalence > 10%)			
Chronic heart disease	60,298 (47.5)	6159 (50.5)	54,139 (47.2)
Chronic kidney disease	22,210 (17.5)	3213 (26.3)	18,997 (16.6)
Mood disorders	18,932 (14.9)	2068 (16.9)	16,864 (14.7)
Arthropathy and arthritis	18,348 (14.5)	1722 (14.11)	16,626 (14.5)
Solid malignancy	18,045 (14.2)	2274 (18.6)	15,771 (13.8)
Esophageal disorders	17,864 (14.1)	2014 (16.5)	15,850 (13.8)
Other nervous system disorders	16,349 (12.9)	1952 (16.0)	14,397 (12.6)
Chronic obstructive pulmonary disease and bronchiectasis	14,696 (11.6)	1902 (15.6)	12,793 (11.2)
Thyroid disorders	14,640 (11.5)	1564 (12.8)	13,076 (11.4)
Substance-related disorders	12,863 (10.1)	1204 (9.9)	11,659 (10.2)
Hospitalization characteristics			
Length of stay, days	5 (3, 8)	5 (3, 9)	4 (3, 8)
Number of admissions in the past year	0 (0, 2)	2 (0, 4)	0 (0, 2)

Data are N (%) or median with interquartile range

### Number of diseases and odds for readmission

**Fig. 1:**
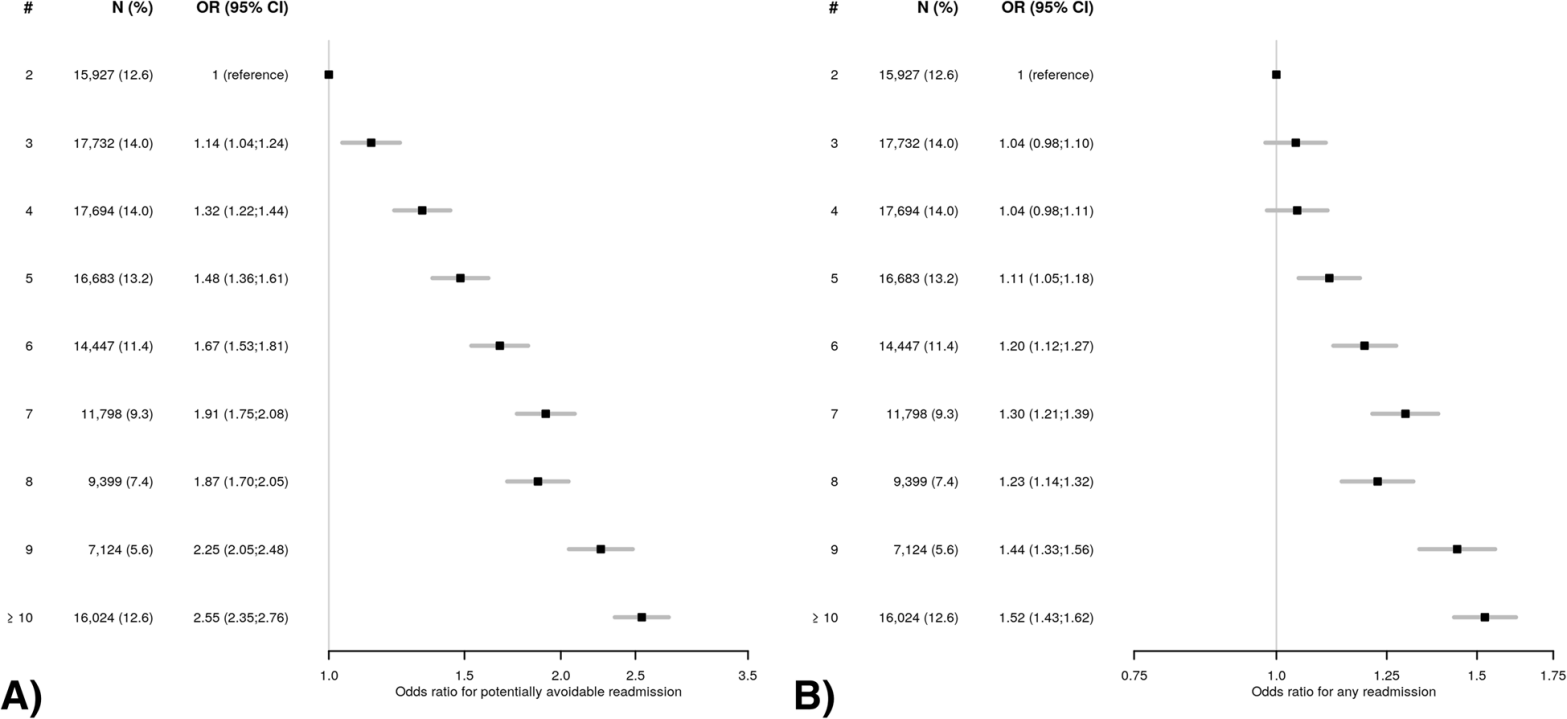
Odds ratios with 95% confidence intervals for **a**) potentially avoidable readmission and **b**) all-cause readmission according to the number of chronic diseases. The reference is the presence of two chronic diseases, as we included only patients with multimorbidity. Abbreviations: #, number of chronic diseases; CI, confidence interval; N, number of patients; OR, odds ratio

The OR for readmission progressively and linearly increased with the number of diseases to up to 2.55 (95%CI 2.35–2.76) for PAR and 1.52 (95%CI 1.43–1.62) for ACR in patients with ten or more diseases, compared to two diseases (Fig. 1). The odds for PAR already more than doubled in the presence of nine diseases (OR 2.25, 95%CI 2.05–2.48).

### Number of body system categories and odds for readmission

**Fig. 2:**
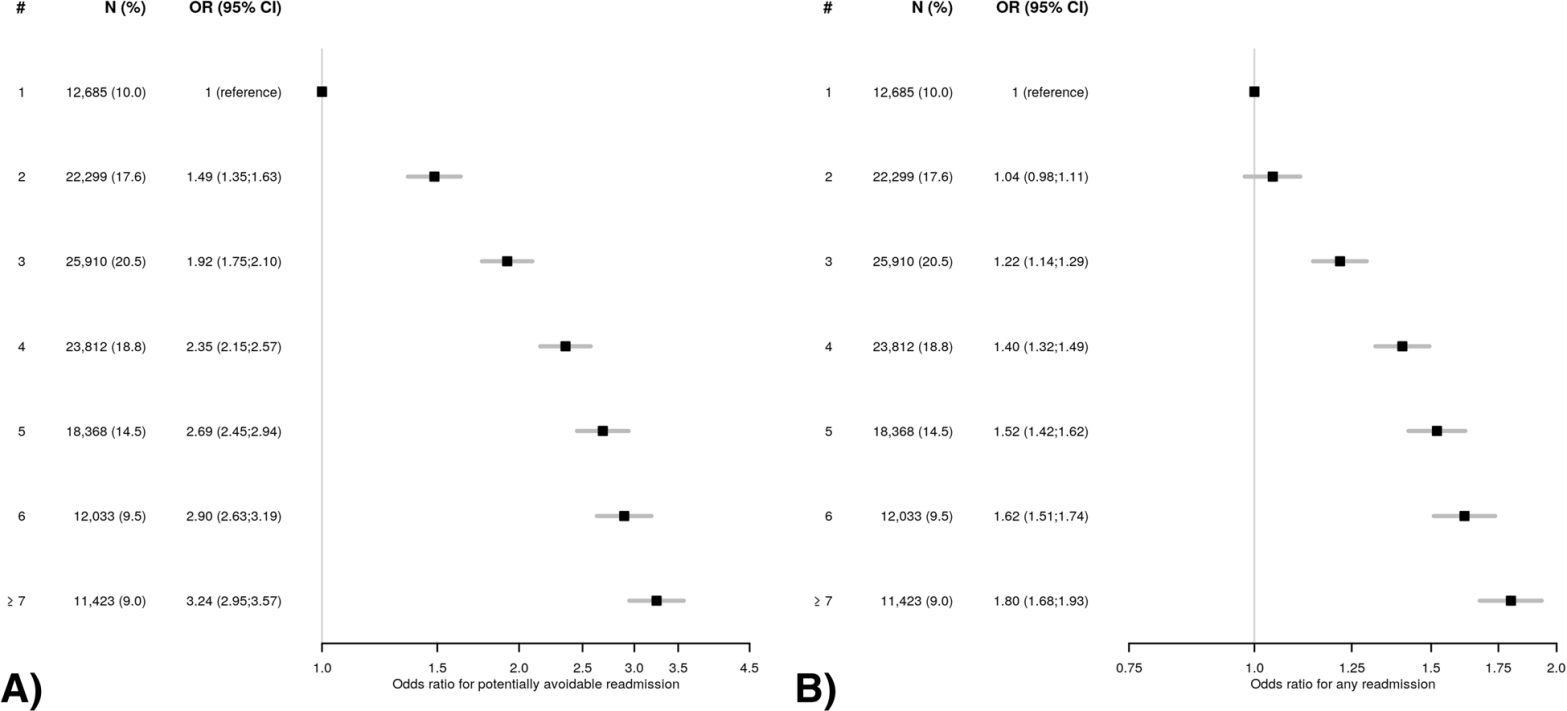
Odds ratios with 95% confidence intervals for **a**) potentially avoidable readmission and **b**) all-cause readmission according to the number of body systems involved. Abbreviations: #, number of body systems; CI, confidence interval; N, number of patients; OR, odds ratio

The OR for readmission progressively and linearly increased with the number of body system categories to up to 3.24 (95%CI 2.95–3.57) for PAR and 1.80 (95%CI 1.68–1.93) for ACR in patients with seven or more body systems involved, compared to one body system involved (Fig. 2). The odds for PAR already more than doubled in the presence of four body systems involved (OR 2.35, 95%CI 2.15–2.57).

### Combinations of diseases categories and odds for PAR

Table 2 presents the 20 combinations of diseases categories with the highest OR for PAR and the interactions between the diseases categories. The odds increased by 74 to 136%. Among those 20 combinations, urogenital diseases, including chronic kidney disease, were most frequent, while chronic ulcer of skin was found in one fifth of the combinations. The highest OR (2.36, 95%CI 2.05–2.71) was found for chronic kidney disease combined with liver disease, followed by chronic kidney disease combined with chronic ulcer of skin (OR 2.18, 95% CI 1.95–2.45). The odds for PAR doubled for chronic heart disease combined with other diseases of kidney and ureters, as well as for chronic kidney disease combined with substance-related disorders or with other diseases of kidney and ureters. Three combinations of diseases categories (chronic kidney disease with substance-related disorders; paralysis with chronic ulcer of skin; esophageal disorders with liver disease) had a more than multiplicative effect on the odds for PAR, and four combinations (chronic kidney disease with other nutritional, endocrine or metabolic disorders; chronic kidney disease with nephritis, nephrosis, renal sclerosis; chronic kidney disease with chronic obstructive pulmonary disease and bronchiectasis; chronic kidney disease with pulmonary heart disease) had a less than multiplicative effect on the odds for PAR.

**Table 2:** Twenty combinations of diseases categories with the highest odds ratio for potentially avoidable readmission, in comparison with patients without the combination, and the interactions between the diseases categories

Chronic disease 1	Chronic disease 2	OR (95% CI)	Interaction
Chronic kidney disease	Liver disease	2.36 (2.05;2.71)	0
Chronic kidney disease	Chronic ulcer of skin	2.18 (1.95;2.45)	0
Chronic heart disease	Other diseases of kidney and ureters	2.02 (1.75;2.34)	0
Chronic kidney disease	Substance-related disorders	2.00 (1.73;2.32)	+
Chronic kidney disease	Other diseases of kidney and ureters	1.99 (1.76;2.26)	0
Chronic kidney disease	Other nutritional, endocrine or metabolic disorders	1.93 (1.63;2.27)	–
Chronic kidney disease	Asthma	1.90 (1.64;2.19)	0
Chronic ulcer of skin	Liver disease	1.89 (1.60;2.23)	0
Chronic kidney disease	Epilepsy; convulsions	1.87 (1.56;2.24)	0
Chronic kidney disease	Nephritis, nephrosis, renal sclerosis	1.87 (1.66;2.09)	–
Paralysis	Chronic ulcer of skin	1.86 (1.55;2.22)	+
Chronic kidney disease	Mood disorders	1.83 (1.65;2.02)	0
Solid malignancy	Asthma	1.82 (1.51;2.18)	0
Chronic kidney disease	Chronic obstructive pulmonary disease and bronchiectasis	1.78 (1.62;1.96)	–
Peripheral and visceral atherosclerosis	Chronic ulcer of skin	1.78 (1.45;2.17)	0
Esophageal disorders	Liver disease	1.76 (1.54;2.01)	+
Chronic heart disease	Nephritis, nephrosis, renal sclerosis	1.76 (1.54;2.00)	0
Chronic kidney disease	Pulmonary heart disease	1.74 (1.56;1.94)	–
Other nervous system disorders	Pulmonary heart disease	1.74 (1.47;2.06)	0
Mood disorders	Liver disease	1.74 (1.47;2.05)	0

*Abbreviations*: *CI* confidence interval, *OR* odds ratio

The signs “+” and “-” represent diseases categories with a significant more than multiplicative effect and less than multiplicative effect on the odds for readmission, respectively. A more than multiplicative effect means that the two diseases categories in combination increase the odds for readmission more than just by multiplying the odds for readmission of each disease category separately. The sign “0” means that the interaction is not significant

### Combinations of diseases categories and odds for ACR

Table 3 displays the 20 combinations of diseases categories with the highest OR for ACR and the interactions between the diseases categories. The odds increased by 63 to 213%. A hematological malignancy was found among the seven combinations with the highest OR, with a maximal OR when combined with esophageal disorders (OR 3.13, 95% CI 2.79–3.52). Chronic kidney disease was the most frequent disease found in the following 13 combinations with the highest OR. In terms of interaction effect between the diseases categories, five combinations had a less than multiplicative effect, and three combinations had a more than multiplicative effect on the odds for ACR.

**Table 3:** Twenty combinations of diseases categories with the highest odds ratio for all-cause readmission, in comparison with patients without the combination, and the interactions between the diseases categories

Chronic disease 1	Chronic disease 2	OR (95% CI)	Interaction
Hematological malignancy	Esophageal disorders	3.13 (2.79;3.52)	0
Hematological malignancy	Mood disorders	2.90 (2.57;3.28)	0
Hematological malignancy	Diseases of white blood cells	2.82 (2.58;3.09)	–
Hematological malignancy	Thyroid disorders	2.48 (2.14;2.88)	0
Hematological malignancy	Other nervous system disorders	2.40 (2.12;2.71)	–
Hematological malignancy	Arthropathy and arthritis	2.23 (1.90;2.61)	0
Hematological malignancy	Chronic heart disease	2.18 (1.99;2.39)	–
Chronic kidney disease	Liver disease	2.03 (1.79;2.32)	0
Solid malignancy	Asthma	1.84 (1.58;2.15)	0
Chronic heart disease	Other diseases of kidney and ureters	1.84 (1.62;2.10)	0
Chronic kidney disease	Chronic ulcer of skin	1.84 (1.66;2.04)	0
Solid malignancy	Substance-related disorders	1.812(1.58;2.08)	0
Hematological malignancy	Chronic kidney disease	1.80 (1.54;2.10)	–
Liver disease	Chronic ulcer of skin	1.78(1.53;2.07)	0
Chronic kidney disease	Other diseases of kidney and ureters	1.73 (1.55;1.93)	0
Esophageal disorders	Liver disease	1.72 (1.54;1.92)	+
Chronic kidney disease	Substance-related disorders	1.70 (1.49;1.93)	+
Solid malignancy	Diseases of white blood cells	1.69 (1.50;1.91)	–
Chronic kidney disease	Diseases of white blood cells	1.67 (1.41;1.99)	0
Chronic kidney disease	Epilepsy; convulsions	1.63 (1.38;1.93)	+

*Abbreviations*: *CI* confidence interval, *OR* odds ratio

The signs “+” and “-” represent diseases categories with a significant more than multiplicative effect and less than multiplicative effect on the odds for readmission, respectively. A more than multiplicative effect means that the two diseases categories in combination increase the odds for readmission more than just by multiplying the odds for readmission of each disease category separately. The sign “0” means that the interaction is not significant

## Discussion

In this large multinational retrospective cohort of multimorbid medical inpatients, we found a strong and linear association of 30-day PAR with the number of body systems involved, and to a lesser extend with the number of chronic diseases. Having four body systems involved or nine chronic diseases already more than doubled the risk for PAR. The number of body systems may therefore be an interesting measure of the risk for readmission in multimorbid patients. The combinations of diseases categories with the strongest association with 30-day PAR included chronic kidney disease with liver disease or with chronic ulcer of skin, and chronic heart disease with other diseases of kidney and ureters. For ACR, the strongest associations were found for a hematological malignancy combined with esophageal disorders, with mood disorders or with diseases of white blood cells.

Consistent with our findings, a few studies had described a positive association between multimorbidity and readmission in medical patients or Medicare beneficiaries, but none had assessed specific patterns of multimorbidity [7, 10, 11]. Furthermore, in this study, unlike previous authors, we separately assessed the outcomes of PAR and ACR. This distinction allowed us to uncover two relevant points. First, greater multimorbidity and similar combinations of diseases categories were more strongly associated with PAR than with ACR. Second, the combinations with the strongest association with PAR or with ACR included different categories of diseases.

While a hematological or a solid malignancy were frequent among the 20 combinations with the highest odds for ACR, we found neither a hematological nor a solid malignancy among the combinations with the highest odds for PAR. This suggests that hospitalizations related to malignancy were for planned oncologic therapy rather than for treatment complications that would have appeared in relationship with PAR also, and not only with ACR. In contrast, combinations with the highest odds for PAR most often included chronic kidney disease. Whereas repeated hospitalizations for planned oncologic treatment are unavoidable, we may nonetheless influence the rate of hospitalization related to diseases affecting the urogenital tract. Describing which combinations of diseases categories are associated with higher odds for PAR specifically, rather than for ACR, may therefore help to identify situations of vulnerability that should be detected early in order to focus those efficient preventive interventions on higher-risk patients.

The high frequency of chronic kidney disease among the combinations with the strongest association with PAR suggests that patients with chronic kidney disease are particularly affected by adverse consequences of multimorbidity, especially higher healthcare resource utilization. This might be due to the high number of complications related to chronic kidney disease, such as bone disease, coagulation disturbances, anemia or cardiovascular diseases. Interestingly, when looking at combinations most strongly associated with ACR after excluding hematological and solid malignancy, eight of the nine combinations were also found among the 20 top combinations associated with PAR, and included mostly chronic kidney disease. This suggests that these associations found for AR were related to PAR rather than to unavoidable readmissions, and underlines again the target group for preventive interventions represented by patients with chronic kidney disease.

We found higher OR for ACR than for PAR. At a first sight, this may seem inconsistent with the stronger relationship with PAR when assessing multimorbidity as a count of diseases or of body systems involved. However, when comparing the results for the same combinations of diseases categories, the OR for PAR was higher than for ACR. We can thus explain the higher OR for ACR than for PAR by the fact that the seven top combinations of diseases with the highest OR for ACR increased the odds for unavoidable rather than for avoidable readmission, which are both included in the composite outcome of ACR. While many studies described frequent combinations of diseases, we found no data assessing their association with readmission that could be compared with our results [2, 3, 16–23].

Previous analyses showed that the burden of multimorbidity increased with each additional disease [7, 8]. In Medicare beneficiaries, the rate of readmission was indeed about 12% in the presence of 0 or 1 chronic condition, and 30% in the presence of six or more chronic conditions, respectively [7]. However, this analysis was restricted to 15 chronic conditions selected from the CMS Chronic Conditions Warehouse (CWS) and to Medicare patients only, and categorized broadly the numbers of chronic conditions (0–1, 2–3, 4–5, 6 or more), while we did not limit our analysis to Medicare patients. Until now, little was known about the cutoffs at which the odds for readmission doubles. Furthermore, we lacked data on how the different diseases may interact together to influence the odds for readmission, i.e. whether the odds associated with each disease just multiply, or if sometimes more or less than multiplicative effects may exist. We therefore assessed interactions between combinations of diseases categories to uncover potentially more complex effects on the odds for readmission. Among the 20 combinations of diseases categories with the highest OR for PAR or ACR, we found that more than one third of the diseases significantly interacted together, most often negatively, corresponding to a less than multiplicative effect on the odds for readmission, and less often positively, corresponding to a more than multiplicative effect on the odds.

These various patterns of interactions, as well as the stronger association with the number of body system involved than with the number of diseases, support the fact that multimorbidity is a complex concept and that measuring it simply as a count of diseases may not be accurate enough and mask important information on the exact risk associated with particular combinations of diseases [12, 13]. A refined and standardized definition of multimorbidity taking this consideration into account might be useful.

### Strengths and limitations

Our study presents some limitations. First, we included only readmissions to the same medical center. Therefore, we cannot exclude to have missed some readmissions to other medical hospitals. Second, as we wanted to focus on multimorbidity of medical patients, our results may not be generalizable to other patients’ population such as surgical patients. Third, although we could assess a broad number of diseases using ICD codes, some diagnoses may not have been coded, so that we cannot exclude some underreporting. Finally, the restriction of our analysis to chronic diseases may have prevented comparison with other studies that also included risk factors and complications of diseases.

This study has a number of strengths also. First, this is the first such study using a large, multinational and multicenter sample of medical inpatients, increasing results’ generalizability. Second, we included a large number of diseases and assessed multimorbidity with standardized classification tools that allow reproducibility [14, 15]. Third, we studied the association of readmission with multimorbidity in several ways, using the total count of diseases, the number of body systems involved, as well as combinations of diseases categories and the interactions between the diseases categories. Finally, unlike previous studies, we distinguished PAR and ACR, which allowed uncover unknown and clinically relevant differences.

## Conclusions

In a large cohort of multimorbid medical inpatients, we found that the odds for 30-day PAR and ACR increased progressively and linearly with the number of body systems involved and with the number of chronic diseases. Chronic kidney disease was almost constantly present in the combinations of diseases categories with the highest OR for PAR. The odds for PAR more than doubled in the presence of four body systems involved. The number of body systems involved may represent an interesting, simple and useful way to assess the risk for readmission in multimorbid medical patients. The identification of combination of diseases with a higher risk for PAR specifically is of particular relevance because it may help to target preventive interventions to high-risk patients most likely to benefit.

## Additional file

Additional file 1: This article has an additional file, which contains following details on the methods: 1) The list of the 18 body system categories of the Chronic Condition Indicator. 2) Details on categorization of diseases, i.e. the complete list of CCS categories found in the patients, with the number of the respective categories according to CCS classification, and the details on CCS categories merged into broader categories. 3) The additional Figure S1, showing the study flow-chart. (DOCX 52 kb)

## Data Availability

The datasets used and/or analysed during the current study are available from the corresponding author on reasonable request (caroleelodie.aubert@insel.ch).

## Abbreviations

ACR: All-cause readmission
CCI: Chronic Condition Indicator
CCS: Clinical Classification Software
CI: confidence interval
ICD: International Classification of Diseases
IQR: interquartile range
IRB: Institutional Review Board
OR: Odds ratio
PAR: Potentially avoidable readmission
