# Supplementary material for: Patterns of multimorbidity associated with 30-day readmission: a multinational study
Source: BMC Public Health. 2019 Jun 13;19:738. doi: 10.1186/s12889-019-7066-9 (PMC6567629; doi:10.1186/s12889-019-7066-9)

**Patterns of multimorbidity associated with 30-day readmission:**

A multinational study

**ADDITIONAL FILE**

This Additional File contains details on the methods and a flow-chart of the study.

**Eighteen body system categories of the Chronic Condition Indicator**

The Chronic Condition Indicator classifies the chronic diseases into following 18 body system categories:

1. infectious and parasitic diseases;
2. neoplasms;
3. endocrine, nutritional and metabolic diseases, and immunity disorders;
4. diseases of blood and blood-forming organs;
5. mental disorders;
6. diseases of the nervous system and sense organs;
7. diseases of the circulatory system;
8. diseases of the respiratory system;
9. diseases of the digestive system;
10. diseases of the genitourinary system;
11. complications of pregnancy, childbirth, and the puerperium;
12. diseases of the skin and subcutaneous tissue;
13. diseases of the musculoskeletal system;
14. congenital anomalies;
15. certain conditions originating in the perinatal period;
16. symptoms, signs, and ill-defined conditions;
17. injury and poisoning;
18. factors influencing health status and contact with health services.

**Categorization of diseases**

Complete list of Clinical Classification Software (CCS) categories found in the patients, with the number of the category, according to CCS classification:

| 1 | Tuberculosis |
| --- | --- |
| 2 | Septicemia (except in labor) |
| 3 | Bacterial infection; unspecified site |
| 4 | Mycoses |
| 5 | HIV infection |
| 6 | Hepatitis |
| 7 | Viral infection |
| 8 | Other infections; including parasitic |
| 9 | Sexually transmitted infections (not HIV or hepatitis) |
| 10 | Immunizations and screening for infectious disease |
| 11 | Cancer of head and neck |
| 12 | Cancer of esophagus |
| 13 | Cancer of stomach |
| 14 | Cancer of colon |
| 15 | Cancer of rectum and anus |
| 16 | Cancer of liver and intrahepatic bile duct |
| 17 | Cancer of pancreas |
| 18 | Cancer of other GI organs; peritoneum |
| 19 | Cancer of bronchus; lung |
| 20 | Cancer; other respiratory and intrathoracic |
| 21 | Cancer of bone and connective tissue |
| 22 | Melanomas of skin |
| 23 | Other non-epithelial cancer of skin |
| 24 | Cancer of breast |
| 25 | Cancer of uterus |
| 26 | Cancer of cervix |
| 27 | Cancer of ovary |
| 28 | Cancer of other female genital organs |
| 29 | Cancer of prostate |
| 30 | Cancer of testis |
| 31 | Cancer of other male genital organs |
| 32 | Cancer of bladder |
| 33 | Cancer of kidney and renal pelvis |
| 34 | Cancer of other urinary organs |
| 35 | Cancer of brain and nervous system |
| 36 | Cancer of thyroid |
| 37 | Hodgkin`s disease |
| 38 | Non-Hodgkin`s lymphoma |
| 39 | Leukemias |
| 40 | Multiple myeloma |
| 41 | Cancer; other and unspecified primary |
| 42 | Secondary malignancies |
| 43 | Malignant neoplasm without specification of site |
| 44 | Neoplasms of unspecified nature or uncertain behavior |
| 46 | Benign neoplasm of uterus |
| 47 | Other and unspecified benign neoplasm |
| 48 | Thyroid disorders |
| 49 | Diabetes mellitus without complication |
| 50 | Diabetes mellitus with complications |
| 51 | Other endocrine disorders |
| 52 | Nutritional deficiencies |
| 53 | Disorders of lipid metabolism |
| 54 | Gout and other crystal arthropathies |
| 55 | Fluid and electrolyte disorders |
| 56 | Cystic fibrosis |
| 57 | Immunity disorders |
| 58 | Other nutritional; endocrine; and metabolic disorders |
| 59 | Deficiency and other anemia |
| 60 | Acute posthemorrhagic anemia |
| 61 | Sickle cell anemia |
| 62 | Coagulation and hemorrhagic disorders |
| 63 | Diseases of white blood cells |
| 64 | Other hematologic conditions |
| 76 | Meningitis (except that caused by tuberculosis or sexually transmitted disease) |
| 77 | Encephalitis (except that caused by tuberculosis or sexually transmitted disease) |
| 78 | Other CNS infection and poliomyelitis |
| 79 | Parkinson`s disease |
| 80 | Multiple sclerosis |
| 81 | Other hereditary and degenerative nervous system conditions |
| 82 | Paralysis |
| 83 | Epilepsy; convulsions |
| 84 | Headache; including migraine |
| 85 | Coma; stupor; and brain damage |
| 86 | Cataract |
| 87 | Retinal detachments; defects; vascular occlusion; and retinopathy |
| 88 | Glaucoma |
| 89 | Blindness and vision defects |
| 90 | Inflammation; infection of eye (except that caused by tuberculosis or sexually transmitteddisease) |
| 91 | Other eye disorders |
| 92 | Otitis media and related conditions |
| 93 | Conditions associated with dizziness or vertigo |
| 94 | Other ear and sense organ disorders |
| 95 | Other nervous system disorders |
| 96 | Heart valve disorders |
| 97 | Peri-; endo-; and myocarditis; cardiomyopathy (except that caused by tuberculosis or sexually transmitted disease) |
| 98 | Essential hypertension |
| 99 | Hypertension with complications and secondary hypertension |
| 100 | Acute myocardial infarction |
| 101 | Coronary atherosclerosis and other heart disease |
| 102 | Nonspecific chest pain |
| 103 | Pulmonary heart disease |
| 104 | Other and ill-defined heart disease |
| 105 | Conduction disorders |
| 106 | Cardiac dysrhythmias |
| 107 | Cardiac arrest and ventricular fibrillation |
| 108 | Congestive heart failure; nonhypertensive |
| 109 | Acute cerebrovascular disease |
| 110 | Occlusion or stenosis of precerebral arteries |
| 111 | Other and ill-defined cerebrovascular disease |
| 112 | Transient cerebral ischemia |
| 113 | Late effects of cerebrovascular disease |
| 114 | Peripheral and visceral atherosclerosis |
| 115 | Aortic; peripheral; and visceral artery aneurysms |
| 116 | Aortic and peripheral arterial embolism or thrombosis |
| 117 | Other circulatory disease |
| 118 | Phlebitis; thrombophlebitis and thromboembolism |
| 119 | Varicose veins of lower extremity |
| 120 | Hemorrhoids |
| 121 | Other diseases of veins and lymphatics |
| 122 | Pneumonia (except that caused by tuberculosis or sexually transmitted disease) |
| 123 | Influenza |
| 124 | Acute and chronic tonsillitis |
| 125 | Acute bronchitis |
| 126 | Other upper respiratory infections |
| 127 | Chronic obstructive pulmonary disease and bronchiectasis |
| 128 | Asthma |
| 129 | Aspiration pneumonitis; food/vomitus |
| 130 | Pleurisy; pneumothorax; pulmonary collapse |
| 131 | Respiratory failure; insufficiency; arrest (adult) |
| 132 | Lung disease due to external agents |
| 133 | Other lower respiratory disease |
| 134 | Other upper respiratory disease |
| 135 | Intestinal infection |
| 136 | Disorders of teeth and jaw |
| 137 | Diseases of mouth; excluding dental |
| 138 | Esophageal disorders |
| 139 | Gastroduodenal ulcer (except hemorrhage) |
| 140 | Gastritis and duodenitis |
| 141 | Other disorders of stomach and duodenum |
| 142 | Appendicitis and other appendiceal conditions |
| 143 | Abdominal hernia |
| 144 | Regional enteritis and ulcerative colitis |
| 145 | Intestinal obstruction without hernia |
| 146 | Diverticulosis and diverticulitis |
| 147 | Anal and rectal conditions |
| 148 | Peritonitis and intestinal abscess |
| 149 | Biliary tract disease |
| 151 | Other liver diseases |
| 152 | Pancreatic disorders (not diabetes) |
| 153 | Gastrointestinal hemorrhage |
| 154 | Noninfectious gastroenteritis |
| 155 | Other gastrointestinal disorders |
| 156 | Nephritis; nephrosis; renal sclerosis |
| 157 | Acute and unspecified renal failure |
| 158 | Chronic kidney disease |
| 159 | Urinary tract infections |
| 160 | Calculus of urinary tract |
| 161 | Other diseases of kidney and ureters |
| 162 | Other diseases of bladder and urethra |
| 163 | Genitourinary symptoms and ill-defined conditions |
| 164 | Hyperplasia of prostate |
| 165 | Inflammatory conditions of male genital organs |
| 166 | Other male genital disorders |
| 167 | Nonmalignant breast conditions |
| 168 | Inflammatory diseases of female pelvic organs |
| 169 | Endometriosis |
| 170 | Prolapse of female genital organs |
| 171 | Menstrual disorders |
| 172 | Ovarian cyst |
| 173 | Menopausal disorders |
| 174 | Female infertility |
| 175 | Other female genital disorders |
| 177 | Spontaneous abortion |
| 178 | Induced abortion |
| 179 | Postabortion complications |
| 181 | Other complications of pregnancy |
| 182 | Hemorrhage during pregnancy; abruptio placenta; placenta previa |
| 183 | Hypertension complicating pregnancy; childbirth and the puerperium |
| 184 | Early or threatened labor |
| 185 | Prolonged pregnancy |
| 186 | Diabetes or abnormal glucose tolerance complicating pregnancy; childbirth; or the puerperium |
| 187 | Malposition; malpresentation |
| 188 | Fetopelvic disproportion; obstruction |
| 189 | Previous C-section |
| 190 | Fetal distress and abnormal forces of labor |
| 191 | Polyhydramnios and other problems of amniotic cavity |
| 192 | Umbilical cord complication |
| 193 | OB-related trauma to perineum and vulva |
| 195 | Other complications of birth; puerperium affecting management of mother |
| 196 | Other pregnancy and delivery including normal |
| 197 | Skin and subcutaneous tissue infections |
| 198 | Other inflammatory condition of skin |
| 199 | Chronic ulcer of skin |
| 200 | Other skin disorders |
| 201 | Infective arthritis and osteomyelitis (except that caused by tuberculosis or sexually transmitted disease) |
| 202 | Rheumatoid arthritis and related disease |
| 203 | Osteoarthritis |
| 204 | Other non-traumatic joint disorders |
| 205 | Spondylosis; intervertebral disc disorders; other back problems |
| 206 | Osteoporosis |
| 207 | Pathological fracture |
| 208 | Acquired foot deformities |
| 209 | Other acquired deformities |
| 210 | Systemic lupus erythematosus and connective tissue disorders |
| 211 | Other connective tissue disease |
| 212 | Other bone disease and musculoskeletal deformities |
| 213 | Cardiac and circulatory congenital anomalies |
| 214 | Digestive congenital anomalies |
| 215 | Genitourinary congenital anomalies |
| 216 | Nervous system congenital anomalies |
| 217 | Other congenital anomalies |
| 222 | Hemolytic jaundice and perinatal jaundice |
| 223 | Birth trauma |
| 224 | Other perinatal conditions |
| 225 | Joint disorders and dislocations; trauma-related |
| 226 | Fracture of neck of femur (hip) |
| 227 | Spinal cord injury |
| 228 | Skull and face fractures |
| 229 | Fracture of upper limb |
| 230 | Fracture of lower limb |
| 231 | Other fractures |
| 232 | Sprains and strains |
| 233 | Intracranial injury |
| 234 | Crushing injury or internal injury |
| 235 | Open wounds of head; neck; and trunk |
| 236 | Open wounds of extremities |
| 237 | Complication of device; implant or graft |
| 238 | Complications of surgical procedures or medical care |
| 239 | Superficial injury; contusion |
| 240 | Burns |
| 241 | Poisoning by psychotropic agents |
| 242 | Poisoning by other medications and drugs |
| 243 | Poisoning by nonmedicinal substances |
| 244 | Other injuries and conditions due to external causes |
| 245 | Syncope |
| 246 | Fever of unknown origin |
| 247 | Lymphadenitis |
| 248 | Gangrene |
| 249 | Shock |
| 250 | Nausea and vomiting |
| 251 | Abdominal pain |
| 252 | Malaise and fatigue |
| 253 | Allergic reactions |
| 258 | Other screening for suspected conditions (not mental disorders or infectious disease) |
| 259 | Residual codes; unclassified |
| 650 | Adjustment disorders |
| 651 | Anxiety disorders |
| 652 | Attention-deficit, conduct, and disruptive behavior disorders |
| 653 | Delirium, dementia, and amnestic and other cognitive disorders |
| 654 | Developmental disorders |
| 655 | Disorders usually diagnosed in infancy, childhood, or adolescence |
| 656 | Impulse control disorders, NEC |
| 657 | Mood disorders |
| 658 | Personality disorders |
| 659 | Schizophrenia and other psychotic disorders |
| 660 | Alcohol-related disorders |
| 661 | Substance-related disorders |
| 663 | Screening and history of mental health and substance abuse codes |
| 670 | Miscellaneous mental health disorders |
|  |  |

For clinical relevance, we further merged some of the 285 mutually exclusive categories of the Clinical Classification Software (CCS) into broader categories:

1. chronic heart disease: CCS 105-107 (cardiac dysrhythmias), CCS 100-101 (coronary heart disease), CCS 10 (nonhypertensive congestive heart failure), CCS 96 (heart valve disorder);
2. cerebrovascular diseases: CCS 109 and 111-112;
3. solid malignancies: CCS 11-36 and 41-42;
4. hematological malignancies: CCS 37-40;
5. arthropathy and arthritis: CCS 54 (gout and other crystal arthropathies), CCS 201 (infective arthritis and osteomyelitis), CCS 202 (rheumatoid arthritis and related disease), CCS 203 (osteoarthritis), CCS 204 (other non-traumatic joint disorders), CCS 205 (spondylosis, intervertebral disc disorders and other back problems);
6. osteoporosis and pathological fractures: CCS 206-207;
7. liver disease: CCS 6 and 150-151;
8. psychosis and schizophrenic disorders: CCS 70-71 and 659;
9. other nutritional, endocrine or metabolic disorder: CCS 51 and 58;
10. substance-related disorders: CCS 660-661.

**Additional Figure 1**. Study Flow-Chart.


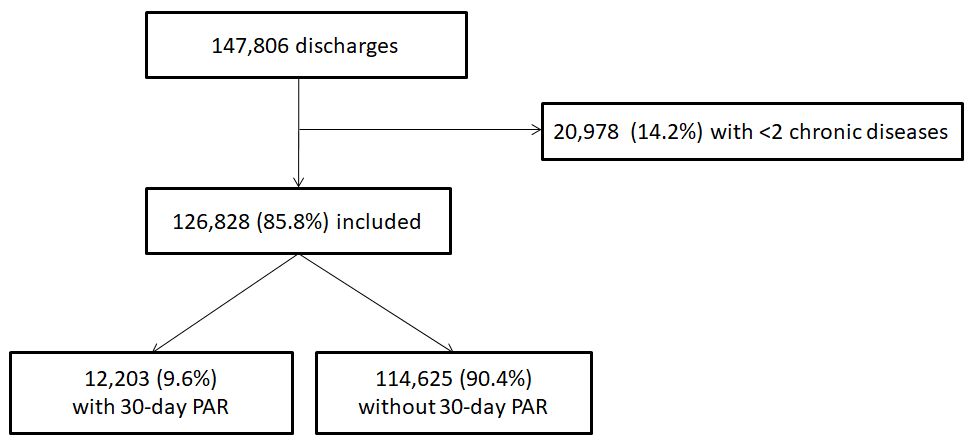

Supplement: Supplementary file 1 — This article has an additional file, which contains following details on the methods: 1) The list of the 18 body system categories of the Chronic Condition Indicator. 2) Details on categorization of diseases, i.e. the complete list of CCS categories found in the patients, with the number of the respective categories according to CCS classification, and the details on CCS categories merged into broader categories. 3) The additional Figure S1, showing the study flow-chart. (DOCX 52 kb) [file 12889_2019_7066_MOESM1_ESM.docx]
